# Microscopic Evaluation of Cleaning Efficiency of Three Different Nickel-Titanium Rotary Instruments

**Published:** 2010-11-15

**Authors:** Maryam Bidar, Saeed Moradi, Maryam Forghani, Salma Bidad, Mahtab Azghadi, Shahrzad Rezvani, Shirin Khoynezhad

**Affiliations:** 1. Department of Endodontics, Dental Research Center of Mashad University of Medical Sciences, Mashhad, Iran.; 2. Endodontist, Mashad, Iran.; 3. Postgraduate Student, Mashad University of Medical Sciences, Mashad, Iran.; 4. General Dentist, Mashad, Iran.; 5. Undergraduate Student, Mashad University of Medical Sciences, Mashad, Iran.

**Keywords:** NiTi Rotary, Root Canal Preparation, Scanning Electron Microscopy, Smear Layer

## Abstract

**INTRODUCTION:**

This study compared the cleaning efficiency of Mtwo, Race and Medin Nickel-Titanium (NiTi) rotary instruments.

**MATERIALS AND METHODS:**

Sixty mandibular molar mesial roots were selected with angle curvatures between 25-35 degrees and divided into three groups; each containing 20 teeth. Canals were prepared with the rotary files and irrigated with 2.5% NaOCl solution after each instrument. Total of 5mL of normal saline was used as the final rinse; subsequently the canals were dried with paper points. The amount of debris and smear layer in three parts of the root canal walls was evaluated using SEM and the data were analyzed by using the Kruskal-Wallis test and the Mann-Whitney U test.

**RESULTS:**

The results for remnant debris in the coronal part of root canals were similar, whereas in the middle third, Mtwo instruments achieved significantly better results compared to Race and Medin instruments. In the apical third of the root canals more debris was created by Race instruments.

**CONCLUSION:**

Overall, Mtwo instruments had greater success in producing clean canals.

## INTRODUCTION

Cleaning and shaping of the root canals creates a smear layer on the canal walls [1]. The smear layer might constitute a source of nutrients for bacterial growth [2]. It may also interfere with the action of irrigants [3] and adhesion and penetration of root canal sealers [4]. When smear layer is not eliminated during root canal treatment, it may disintegrate or be removed by bacterial byproducts allowing leakage [5]. Whether smear layer needs to be removed or retained remains controversial; however, there is growing evidence to support removal of the smear layer before obturation [6][7].

Based on the report of the European Society of Endodontology the main objectives of root canal instrumentation are maintenance of the original canal curvature, elimination of residual pulp tissues and removal of debris [8]. The rotary Nickel-Titanium (NiTi) instruments were developed to serve these purposes and gained rapid acceptance due to advantages of extreme flexibility and increased speed of canal preparation.

According to some reports, rotary files with a flute design may offer some advantages in their cleaning ability. For example, sharp cutting edges seem to be superior to edges that have radial lands in cleaning the root canal [9][10]. One study found Race rotary files removed debris effectively while maintaining the original canal curvature [10].

Schafer et al. compared the shaping ability and cleaning effectiveness of different rotary files and found that Mtwo instrument also gave good results [11].Medin files with taper ratio of 14 %, 10 %, 8 %, 6 %, 4 % and 2 % have been introduced for root canal preparation. The cutting edges of these files are discontinued by grooves in a helix to increase the cutting ability. No previous study has the cleaning ability of Medin rotary files.

The aim of this study was to compare the cleaning efficiency of Mtwo, Race, and Medin rotary files with different blade design in three parts of the canals.

## MATERIALS AND METHODS

Sixty extracted human mandibular molars were selected and the degree of mesial root curvature was determined using a computerized image processing system (Schneider technique) [12]. The teeth with the angle of curvature of 25 to 35 degrees were selected and stored in 5.25% NaOCl for 1 hour to be cleaned. Coronal access was achieved using diamond burs. Only teeth with apical root canal width approximately compatible with size 10 K-file were included. A coronal reference point 14 mm from apical foramen was created by shortening all molar crowns accordingly.

The teeth were randomly divided into three experimental groups of 20 teeth each. The mesial canals were instrumented with Mtwo (VDW, Munich, Germany), Race (FKG Dentaire, La-Chaux-de-fonds,Switzerland) and Medin (MEDIN,a.s. ,Czech Republic) using crown down technique. RcPrep (premier products co, USA) was used as a lubricant during instrumentation and the canals were irrigated with 2.5% NaOCl solution after each instrument. 5mL of normal saline (Samen Industries, Mashad, Iran) was used as final rinse after instrumentation and then the canals were dried.

### Evaluations

Roots were split longitudinally with a diamond disk (D and Z, Diamant 74, Germany) and prepared for evaluation with a scanning electron microscope (SEM;Vega II XMU, Tescan, Czech Republic).

Separate evaluations were recorded for smear layer and remaining debris in three areas of the root canals by means a numerical evaluation scale [13]. The following scale was used.

#### Indices of smear layer dispersion:

**Score 1: **No smear layer, all dentinal tubules open

**Score 2: **Small amount of smear layer, some dentinal tubules open

**Score 3: **Homogeneous smear layer coverage, few dentinal tubules open

**Score 4: **Homogeneous smear layer coverage, no open dentinal tubules

**Score 5: **Thick and inhomogeneous smear layer cover the entire root canal walls

#### Indices of debris dispersion:

**Score 1: **Root canal walls were clean; only few debris particles

**Score 2: **Few conglomerations of debris

**Score 3: **Many conglomerations of debris covered less than 50% of canal walls

**Score 4: **Debris covered more than 50% of canal walls

**Score 5: **Debris covered complete or nearly complete surfaces of canal walls

Scores 1 and 2 of debris and smear layer dispersion were selected as suitable levels of canal cleanliness [14].

Debris and smear layer was rated under a ×500 and ×1500 magnification respectively. The data were statistically analyzed by using the Kruskal-Wallis test and the Mann-Whitney U test, and the significance was set at P= 0.05.

## RESULTS

None of instruments used for canal preparation was able to produce a completely clean canal. The scores of debris and smear layer are presented in table 1 and table 2.

**Table 1:** The scores of remaining debris

** Instruments**	**Coronal third **					**Middle third **					**Apical third**					**Total** ****				
	**1**	**2**	**3**	**4**	**5**	**1**	**2**	**3**	**4**	**5**	**1**	**2**	**3**	**4**	**5**	**1**	**2**	**3**	**4**	**5**
**Mtwo**	10	9	1	0	0	3	8	8	1	0	0	2	10	4	4	13	19	19	5	4
**Medin**	10	10	0	0	0	1	5	14	0	0	0	0	9	7	4	11	15	23	7	4
**Race**	9	10	1	0	0	0	5	10	5	0	0	0	2	10	8	9	15	13	15	8
**P.values**	0.89					0.03					0.01					0.07				

**Table 2:** The scores of smear layer

** Instruments**	**Coronal third **					**Middle third **					**Apical third**					**Total** ****				
	**1**	**2**	**3**	**4**	**5**	**1**	**2**	**3**	**4**	**5**	**1**	**2**	**3**	**4**	**5**	**1**	**2**	**3**	**4**	**5**
**Mtwo**	6	3	9	2	0	2	2	8	7	1	2	2	7	6	3	10	7	24	15	4
**Medin**	6	6	6	2	0	0	1	8	9	2	1	2	7	6	4	7	9	21	17	6
**Race**	4	9	5	2	0	4	0	4	7	5	0	1	9	7	3	8	10	18	16	8
**P.values**	0.85					0.34					0.78					0.68				

**Figure 1:**
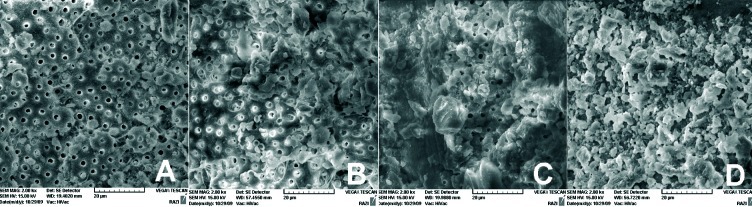
A) Coronal third of the prepared canal with Mtwo files (score 1), B) Middle third of the prepared canal with Medin files (score 3) C) Apical third of canal wall prepared with Race instruments more than 50% covered with debris (score 4) D) Completely covered with debris (score 5).

In the coronal part of the canals, no statistically significant differences were apparent in the remaining debris between experimental groups and 45-50% of specimens having score 1 (Figure 1A).

Mtwo files remained significantly less debris compared with Medin and Race files in the middle third of the canal walls (P<0.05). 50-70% of specimens in Medin and Race files groups showed score 3 (Figure 1B).

In the apical regions, the use of Race files resulted in significantly more residual debris compared with others (P<0.05). 40-50% of specimens in Race files group resulted in score 4 and 5 (Figure 1C).

In terms of smear layer, no significantly differences were apparent between groups (P>0.05) (Table 2). In general the Mtwo files resulted in 28.4%, the Medin instruments in 26.7% and the Race system in 30% of specimens having scores 1 and 2.

6.7%, 10% and 13.3% of specimens in Mtwo, Medin and Race files groups resulted in score 5 respectively (Figure 1D).

## DISCUSSION

One of the most important objectives during root canal preparation is to eliminate microorganisms as much as possible from the root canal system by removing vital and/or necrotic pulp tissue, infected dentin, and debris [15].

The smear layer is a superficial film of dentin particles, and vital or necrotic pulp remnants that are produced when a canal is instrumented [16]. It is considered to be desirable to remove this layer due to its potential deleterious effects [7][17].

Debris was defined as dentin chips, and vital or necrotic pulp remnants loosely attached to the canal walls [13]. There is no doubt that debris removal is a critical issue for elimination of the microorganisms from the root canal system.

The ability to achieve these aims was examined in this experimental study using SEM. SEM has been used in several studies to evaluate the cleaning efficiency of various instrumentation techniques [11][18][19].

The present results indicated that partially uninstrumented areas with remaining debris are found in all experimental groups and canal regions that are in agreement with other studies [11][13]. These findings emphasize the importance of irrigants for effective disinfection of the root canal system [20][21][22].

In order to remove debris and smear layer the use of antibacterial solutions such as sodium hypochlorite is recommended in combination with chelating agents [17][23]. Considering the objective of the present study (to evaluate the cleaning effectiveness of the different rotary files) 2.5% NaOCl alone was used as irrigant to prevent interference of other factors in removing debris and smear layer.

In this study the use of Mtwo files resulted in significantly less remaining debris in the middle third of the canals (Table 1). No significant differences were found between groups in terms of smear layer removal. These results confirm the findings reported by Schafer et al. that found Mtwo instruments remained less debris compared with K3 or Race instruments without significant differences regarding smear layer removal [11]. The authors mentioned that the design of Mtwo files (two sharp cutting edges and a small core diameter) may enhance its debris removal capacity.

The present results indicate that the apical thirds of the canals were less clean than the middle and coronal thirds in all groups that is inconsistent with others [20][21]. Moreover, the use of Race files resulted in significantly more residual debris in the apical third of the canals. Schafer et al. also reported that Race files resulted in more debris compared with Mtwo files [11]. This finding may be important because the effectiveness of irrigants is also reduced as we get closer to the apex [24].

Other studies which evaluate time of canal preparation, canal straightening after preparation and defects of these instruments during instrumentation seems valuable and are suggested.

## CONCLUSION

In conclusion, though all experimental groups demonstrated greater success for Mtwo rotary instruments in removing debris, in terms of smear layer no significant differences were found.

## References

[1] Cohen S, Hargreaves KM (2006). Pathways of the pulp, 9th Edition.

[2] Pashley DH (1984). Smear layer: physiological considerations. Oper Dent Suppl.

[3] Orstavik D, Haapasalo M (1990). Disinfection by endodontic irrigants and dressings of experimentally infected dentinal tubules. Endod Dent Traumatol.

[4] Kouvas V, Liolios E, Vassiliadis L, Parissis-Messimeris S, Boutsioukis A (1998). Influence of smear layer on depth of penetration of three endodontic sealers: an SEM study. Endod Dent Traumatol.

[5] Sen BH, Wesselink PR, Türkün M (1995). The smear layer: a phenomenon in root canal therapy. Int Endod J.

[6] Hülsmann M, Heckendorff M, Lennon A (2003). Chelating agents in root canal treatment: mode of action and indications for their use. Int Endod J.

[7] Shahravan A, Haghdoost AA, Adl A, Rahimi H, Shadifar F (2007). Effect of smear layer on sealing ability of canal obturation: a systematic review and meta-analysis. J Endod.

[8] (1994). European Society of Endodontology. Consensus report of the European Society of Endodontology on quality guidelines for endodontic treatment. Int Endod J.

[9] Jeon IS, Spångberg LS, Yoon TC, Kazemi RB, Kum KY (2003). Smear layer production by 3 rotary reamers with different cutting blade designs in straight root canals: a scanning electron microscopic study. Oral Surg Oral Med Oral Pathol Oral Radiol Endod.

[10] Schäfer E, Vlassis M (2004). Comparative investigation of two rotary nickel-titanium instruments: ProTaper versus RaCe. Part 2. Cleaning effectiveness and shaping ability in severely curved root canals of extracted teeth. Int Endod J.

[11] Schäfer E, Erler M, Dammaschke T (2006). Comparative study on the shaping ability and cleaning efficiency of rotary Mtwo instruments. Part 2. Cleaning effectiveness and shaping ability in severely curved root canals of extracted teeth. Int Endod J.

[12] Schneider SW (1971). A comparison of canal preparations in straight and curved root canals. Oral Surge Oral Med Oral Pathol.

[13] Hülsmann M, Rümmelin C, Schäfers F (1997). Root canal cleanliness after preparation with different endodontic handpieces and hand instruments: a comparative SEM investigation. J Endod.

[14] Suffridge CB, Hartwell GR, Walker TL (2003). Cleaning efficiency of nickel-titanium GT and .04 rotary files when used in a torque-controlled rotary handpiece. J Endod.

[15] Schäfer E, Lohmann D (2002). Efficiency of rotary nickel-titanium FlexMaster instruments compared with stainless steel hand K-Flexofile--Part 2. Cleaning effectiveness and instrumentation results in severely curved root canals of extracted teeth. Int Endod J.

[16] Grandini S, Balleri P, Ferrari M (2002). Evaluation of Glyde File Prep in combination with sodium hypochlorite as a root canal irrigant. J Endod.

[17] Lim TS, Wee TY, Choi MY, Koh WC, Sae-Lim V (2003). Light and scanning electron microscopic evaluation of Glyde File Prep in smear layer removal. Int Endod J.

[18] Zand V, Bidar M, Ghaziani P, Rahimi S, Shahi S (2007). A comparative SEM investigation of the smear layer following preparation of root canals using nickel titanium rotary and hand instruments. J Oral Sci.

[19] Jodway B, Hülsmann M (2006). A comparative study of root canal preparation with NiTi-TEE and K3 rotary Ni-Ti instruments. Int Endod J.

[20] Hülsmann M, Gressmann G, Schäfers F (2003). A comparative study of root canal preparation using FlexMaster and HERO 642 rotary Ni-Ti instruments. Int Endod J.

[21] Paqué F, Musch U, Hülsmann M (2005). Comparison of root canal preparation using RaCe and ProTaper rotary Ni-Ti instruments. Int Endod J.

[22] De-Deus G, Garcia-Filho P (2009). Influence of the NiTi rotary system on the debridement quality of the root canal space. Oral Surg Oral Med Oral Pathol Oral Radiol Endod.

[23] Gambarini G (1999). Shaping and cleaning the root canal system: a scanning electron microscopic evaluation of a new instrumentation and irrigation technique. J Endod.

[24] O'Connell MS, Morgan LA, Beeler WJ, Baumgartner JC (2000). A comparative study of smear layer removal using different salts of EDTA. J Endod.

